# Procedural Memory Deficits in Preschool Children with Developmental Language Disorder in a Spanish-Speaking Population

**DOI:** 10.3390/brainsci14030198

**Published:** 2024-02-22

**Authors:** Soraya Sanhueza, Mabel Urrutia, Hipólito Marrero

**Affiliations:** 1 Facultad de Humanidades y Arte, Universidad de Concepción, Concepción 4070386, Chile; sorayasanhueza@udec.cl; 2 Facultad de Educación, Universidad de Concepción, Concepción 4070386, Chile; 3 Facultad de Psicología y Logopedia, Universidad de La Laguna, 38200 La Laguna, Spain; hmarrero@ull.edu.es

**Keywords:** procedural memory, developmental language disorder (DLD), procedural deficit hypothesis (PDH), language acquisition, serial reaction time (SRT) task

## Abstract

This study aimed to compare procedural learning skills between Spanish-speaking preschool children (ages 4 years to 4 years, 11 months) with developmental language disorder (DLD) and their chronologically matched typically developing (TD) peers. Using the serial reaction time (SRT) task, participants (30 children with DLD and 30 TD children) responded to visual stimuli in a sequenced manner over four blocks, followed by a random order block. The task assessed reaction time (RT) and accuracy. The results showed a significant interaction between group and block for RT and accuracy, with children with DLD exhibiting longer RTs and accuracy deficits across blocks. In contrast, the TD group showed higher RT efficiency and accuracy in the sequential blocks and, as expected, decreased performance in the random block according to the experimental manipulation. Overall, the results of this investigation suggest that there was no implicit learning in the DLD group, as indicated by the SRT task paradigms of procedural memory. These findings align with some aspects of the procedural deficit hypothesis (PDH), which suggests that linguistic deficits in the DLD population may derive from a deficit in sequential learning from the procedural memory system domain in the Spanish context.

## 1. Introduction

Developmental language disorder (DLD) is a persistent condition impacting language acquisition and development that results in substantial challenges in social interactions and educational advancement [1]. This disorder has a global prevalence and is estimated to affect approximately 3% to 7% of children [2,3]. As DLD is a universally recognized diagnosis, it shares commonalities across languages, including grammatical errors [4]. In the case of Spanish, one of its grammatical characteristics is the type of conjugation characterized by irregular and regular patterns, as well as grammatical moods, aspects, and tenses, with specific morphological features [5].

Some authors discuss the nature of grammatical difficulties faced by children with DLD in Spanish [6,7], emphasizing difficulties with verb use; errors in verb tense, especially for the past tense [8]; errors in the assignment of the argument for a verb, particularly in ditransitive verbs or omitting verbal arguments (e.g., la niña rompió *); and problems with nominal concord markers (e.g., *mi casa amarillo*) or grammatical concordance (e.g., *mi gallina si vuelan*). Deficits in implicit grammatical rules can also be seen, for example, as errors or omissions in function words such as determiners, prepositions, or conjunctions (e.g., *Está (en) la mesa*). Difficulties are also present in sentence and grammatical structures, especially relative clauses (e.g., *pásame el libro que está encima del escritorio*). At the morphosyntactic level, one of the most noteworthy problems is the use of clitics in Spanish [7], for example, the use of *lo*/*la* to refer to an object and the use of *le* to refer to a person (e.g., *al niño le regalaron un juguete*, where *le* refers to the child).

According to Freudenthal et al. [7], English grammar is more complex than Spanish in three important aspects: first, in verbal inflection, since English verbs do not show the morphosyntactic richness of Spanish, which has person, number, tense, and mood verb endings, causing English-speaking children with DLD to make more overgeneralizations by paying more attention to the most relevant aspects of the language. Second, question formulation in English changes in morphology and order, especially in the third person singular. Third, the mandatory use of pronouns in English, unlike in Spanish, leads DLD children to use the same verb form for different combinations of person and number, probably without processing the verb and with greater difficulty in integrating the subject into the sentence.

Freudenthal et al. [7] conducted an experiment with Spanish- and English-speaking children with DLD, training a computational sequential learning model that uses prediction errors to identify relevant signals for correct inflectional marking. Their results showed poorer performance in verbal inflection for English-speaking children with DLD compared to Spanish-speaking children with DLD. These results can be explained by the sequential learning approach that could underlie the verbal marking pattern in children with DLD and that favors Spanish-speaking children, according to the authors. However, evidence from Catalan-Spanish DLD children and English-speaking DLD children with statistical word learning tasks found that, regardless of the language type, both DLD groups showed lower performance than their typically developing counterparts [9].

Spanish-speaking children with DLD also face many challenges with a varied and changing linguistic input from a morphosyntactic perspective due to six combinations of person and number in the present tense compared to only two in English. Thus, one common error in Spanish-speaking children with DLD is confusing the use of the third person plural for the singular (e.g., *ellos canta*), where the same cognitive mechanism of greater attention to relevant stimuli operates [6,7]. These results further highlight the need to investigate the sequential learning aspects involved in the procedural memory task in Spanish to study the behavior of children with DLD in this cognitive task.

Recent research has established connections between linguistic impairments and non-linguistic deficits encompassing learning and memory [10,11]. As a result, a shift has occurred from the concept of specific language impairment (SLI) towards a broader perspective that views DLD as a language development disorder encompassing both linguistic and non-linguistic components [1]. Under this view, there are two main perspectives that attempt to explain the causes of DLD, one of which suggests that the disorder is specifically related to aspects of language such as difficulties in vocabulary acquisition or the understanding of grammatical or phonological rules [4], while the other proposes that it is caused by a deficit in non-linguistic processing such as deficits in executive functions, sustained attention, or memory systems [12,13]. However, both perspectives have limitations in explaining the range of linguistic and non-linguistic impairments observed in DLD. 

The procedural deficit hypothesis (PDH) provides a comprehensive explanation considering linguistic and neural aspects to elucidate the observed pattern of deficits in DLD [13,14,15]. According to the PDH, abnormalities in the brain’s procedural memory system, including the basal ganglia, cerebellum, and portions of the parietal and frontal cortices, contribute to the deficits observed in DLD, while declarative learning and memory functions are preserved [14]. Procedural memory underlies various skills, such as sequencing, learning probabilistic rules, categorization, and rule-governed aspects of grammar [13,14,16,17,18]. Within these skills, a considerable amount of the research conducted in the DLD population focuses on the assessment of sequential learning by the experimental paradigm known as the serial reaction time (SRT) task, originally devised by Nissen and Bullemer in 1987 [19].

The SRT task is designed to evaluate how individuals learn patterns without conscious awareness. During the task, participants are presented with a series of visual stimuli and are required to respond swiftly by pressing buttons. As participants encounter the sequence, their reaction times progressively decrease, and their accuracy improves as they unconsciously internalize the pattern. This task’s use as an indicator of procedural memory function is substantiated by prior research demonstrating the integral role of procedural memory structures in task performance [20,21]. 

The generalization of performance on the SRT task to impairments in procedural learning is questionable. Recent evidence suggests that other procedural memory skills remain unaffected in the population of children with DLD, such as motor skill learning [22,23,24]. In studies of procedural memory in adults, research has linked differences in language skills to deficits in motor skill learning, categorization, and learning and priming tasks involving repetitive exposures to the same stimuli. Notably, this association was not found with sequential learning, as measured by SRT [25]. 

Although we could argue that the SRT task does not encompass all domains of procedural memory, robust evidence in the DLD population has linked its performance to language abilities. Studies conducted in the context of English-speaking preschool children at the age of 5 have found differences between DLD and TD populations, revealing that sequence learning reaches a relatively advanced stage of development [26,27]. This developmental aspect can significantly impact the ability to learn and utilize language skills. Individuals with DLD, characterized by deficits in procedural memory, may encounter challenges in acquiring linguistic rules that TD individuals acquire effortlessly and implicitly. Findings in language acquisition have shown that procedural memory impacts the learning of grammatical abilities rather than lexical abilities in both children acquiring their first language and adults learning a second language [28]. 

A meta-analysis by Lum et al. [11] summarized findings from eight studies examining procedural performance in individuals with DLD, using the classic SRT task as their inclusion criterion. The meta-analysis included 186 participants with DLD and 203 TD peers. The findings revealed that the TD group overall demonstrated a greater difference in response times between sequenced and random blocks compared to the DLD group. The meta-analysis yielded an average mean effect size of 0.328, indicating a significant association between DLD and impairments in procedural learning as measured by the SRT task. 

As can be observed, the majority of studies on procedural memory have been conducted in English. Regarding Romance languages, the available evidence can be found in studies conducted in French by Gabriel and colleagues [29,30,31,32], which highlighted discrepancies with the PDH. In a series of experiments, the authors first observed [29] that individuals with DLD performed similarly to controls on an SRT task with an 8-item sequence, which differed from the classical 10-item SRT task. They then investigated the response modality of the SRT task using both a keyboard and a touch screen [30]. Their results indicated that subjects with DLD exhibited learning, and differences from the control group diminished when using the touch screen, suggesting that DLD performance is limited by motor and cognitive demands. In a third experiment, the authors introduced a more complex sequence of 12 items in 6 blocks, followed by a block with a different sequence. In this scenario, they found a learning deficit in DLD children compared to their TD counterparts [31]. Finally, they conducted a comparison between visual and auditory SRT, which revealed similar sequential learning in reaction times between DLD and TD subjects. However, DLD subjects made more errors than controls in the auditory modality [32].

These predominantly contradictory findings from the French language [29,30,32] were observed in a population of children between the ages of 6 and 13 years, an older population in comparison to the population reported in the English studies [14,26,27]. Age has been shown to be a factor influencing performance on the SRT task, with better performance in DLD possibly due to delayed maturation of the procedural memory system, consistent with the PDH [14]. Another factor influencing SRT task performance may be the number of exposures to the sequence, with shorter sequences allowing for more repetition, facilitating sequential learning in children with DLD [8,11]. 

Taking these reports into consideration, we believe it is essential to assess the performance of the SRT task in younger subjects in the context of a Romance language such as Spanish. Evidence from Chilean DLD children aged 5 to 6 years old supports the notion that they are capable of performing tasks characteristic of experimental designs in a go/no-go task designed to assess sentence grammaticality [8]. Overall findings on SRT task performance support that sequential learning is deficient in the DLD population, with more evidence contradicting this in Romance languages [29,30,32]. We hypothesize that the age of the population and the number of elements in the sequence can influence the results, but there is no evidence, to our knowledge, affirming this in another Romance language such as Spanish. 

The implication of procedural memory in DLD has been associated with a potential influence of working memory, since both systems share neural networks (the basal ganglia and their associated circuitry [15] are also associated with language processing [33]). Working memory is a cognitive system responsible for the temporary storage and manipulation of verbal and visual information [34]. Numerous studies have highlighted the role of working memory in various linguistic tasks, including comprehension, sentence construction, and vocabulary acquisition [35,36,37]. 

Although deficits in working memory have been extensively investigated in both verbal and non-verbal tasks among children with DLD, the visual–spatial domain of the working memory system has received comparatively less attention than its verbal counterpart [38]. In a meta-analysis by Vugs et al. [39], the authors conclude that there is a significant impairment in this visual–spatial domain. Longitudinal studies contribute further insights, indicating a slower pattern of development for visual–spatial storage in children with DLD [40]. This variability underscores the complexity of working memory profiles within the visual–spatial domain and emphasizes the need for a more comprehensive understanding in the context of DLD. By introducing working memory as a covariable, we aim to determine whether potential deficits in working memory may contribute to, or interact with, the observed procedural memory differences between children with DLD and their TD peers.

Investigating the differences in procedural learning between native Spanish speakers with DLD and individuals with TD has significant implications. Given that the recommended age for the diagnosis of DLD is 4 years [41] and that the diagnosis is most frequently provided in Chilean educational institutions [42], studying procedural memory in this population could potentially mitigate linguistic limitations. Therefore, this study aims to investigate differences in procedural memory learning between preschool Spanish-speaking children with DLD and their TD peers using the classic SRT task (10 elements) developed by Nissen and Bullemer [19]. The study compares reaction times and the percentage of correct responses to determine whether there are differences in sequential learning involved in procedural memory between the two groups.

## 2. Materials and Methods

The research design is a mixed intergroup 2 (DLD/TD) and intragroup × 5 (blocks: 1, 2, 3, 4, 5) design. Blocks 1 to 4 followed a sequential pattern, whereas block 5 followed a random pattern.

### 2.1. Participants

Thirty children with DLD and 30 TD children participated in the study (see Table 1 for participant characteristics of the final set of children). For the estimation of the minimum required sample size, the following parameters were considered: (a) effect size (f) = 0.25, (b) statistical power (1 − β) = 0.95, (c) significance level (α) = 0.05, and (d) number of measurements = 5. According to these variables, a minimum of 16 individuals per group was needed, as calculated by the G*Power program version 3.1.7. In order to address the school dropout effect, which is common in this educational stage, we considered a sample size almost double that which was required. The children were recruited from schools in Concepción, Chile, and all were native Spanish speakers. Their parents and/or legal guardians provided written consent, and the participants gave their verbal assent before participating in the experiment. This study received ethical approval from the Ethics, Bioethics and Biosafety Committee (Protocol No. CEBB 731-2020) of the University of Concepción, Chile.

Inclusion criteria required that all children be between the ages of 4 years and 4 years, 11 months at the time of the evaluation and that they attend an educational center. All of them had normal or corrected-to-normal vision. The children in the DLD group had previously been diagnosed with the standard assessment instruments required by Chilean ministerial regulations—Teprosif-R, Tecal, and STSG [43]—by a speech–language pathologist and were enrolled in a special language school. 

As the evaluation instruments stipulated by educational regulations cannot be replicated within a 6-month timeframe, we utilized the Test of Initial Language Development (TELD-3: S) to confirm the linguistic diagnosis of the subjects, administered by a single speech–language pathologist. This test is an instrument by the authors Ramos et al. [44] that evaluates the receptive and comprehensive language of subjects aged 2 years to 7 years, 11 months old. The receptive subtest consists of 37 items, with 24 semantic items and 13 grammar items measuring the child’s proficiency in understanding spoken language. Items for the preschool stage include prompts such as “*Show me the car/Muéstrame el auto*” or “*Show me the ball/Muéstrame la pelota*”. As the difficulty level increases, children are prompted with more complex tasks like “*Show me the boy that is under the table/Muéstrame al niño que está abajo de la mesa*”. Towards the end of the test, participants are presented with questions like “*What goes with the word ‘girl’: his or hers?/¿Qué va con la palabra ‘niña’: suyo o suya?*” and “*Tell me if the following words mean the same or mean something different: box, ark/Dime si las siguientes palabras significan lo mismo o significan algo diferente: caja, arca*”.

The expressive subtest comprises 21 semantic and 18 grammar items. These items involve tasks such as sentence repetition and responding to questions like “*What is the kid doing?/¿Qué está haciendo el niño?*” and “*How old are you?/¿Cuántos años tienes?*”. Towards the end of the test, participants are prompted with items like “*Tell me the word to finish the sentence/Dime la palabra necesaria para terminar la oración*” and “*Make a sentence with the words: see-dog/Elabora una oración con las palabras: ver-perro*”. The test, validated in Chile for the diagnosis of DLD, obtained a Cronbach’s alpha of 0.931 in the receptive subtest, 0.947 in the expressive subtest, and 0.969 in the total test. Based on the results of the TELD-3: S test, the two study groups were formed. Children with DLD scored at or below the 10th percentile, while children with TD scored at or above the 25th percentile (see Table 1 for participant scores).

In terms of exclusion criteria, none of the participants in the sample had any known sensory or developmental disorders, including autism, cognitive deficits, and cerebral palsy. In addition, none of the participants had a history of trauma requiring medical attention. This information was specifically gathered in the DLD group through the anamnesis answered by parents or guardians.

### 2.2. Stimuli and Procedure

#### 2.2.1. SRT Task

The SRT task has been employed to investigate procedural learning in various clinical populations. In this task, participants are required to press a button according to the location of a visual stimulus presented on a computer screen. Unknown to the participants, the location of the stimulus follows a predetermined sequence. Learning is deemed to have taken place if participants exhibit quicker responses to stimuli presented in a sequence than to those presented randomly.

**Figure 1:**
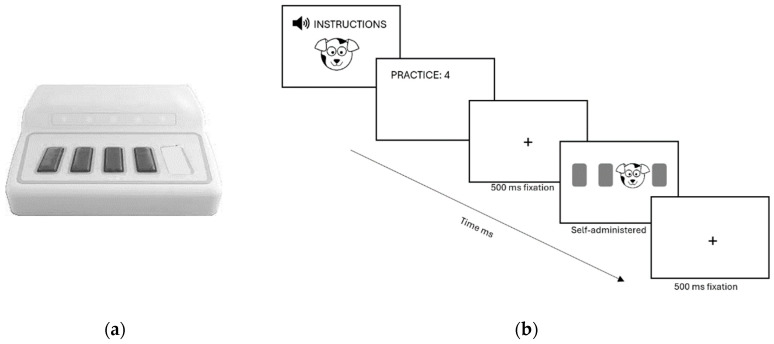
(**a**) Chronos device. (**b**) Presentation stimuli.

The stimuli were presented electronically using E-Prime 3.0 software [45]. A dog was chosen as the target. Each participant received visually supported verbal instructions for the task: “Hello, in this experiment we need your help to find a puppy, it can appear in one of these four positions, when you see it on the screen press the corresponding key on the keypad, you have to do it as fast as possible”. For the response, we utilized the USB response device Chronos [46], employing four of the five horizontally positioned buttons that correspond in shape and color to those displayed on the screen (see Figure 1a). To familiarize the participants with the task, four practice stimuli were presented, followed immediately by the experimental phase (see Figure 1b).

The dog’s appearance was programmed in four different horizontal positions (1-2-3-4). In blocks 1 to 4, the dog appeared in a predetermined sequence of 10 positions (4-2-3-1-3-2-4-3-2), which was repeated six times, resulting in a total of 60 stimuli per block. In block 5, a pseudorandom form was employed, in which each position appeared the same number of times as in the previous blocks, but the probability of the next location was controlled to avoid coinciding with the serial pattern.

After each block, the participants were given a break. Once they were ready to resume the experiment, they were asked to press a button to continue. At the end of the experiment, the participants received an auditory congratulations and were shown an image of a trophy. The total duration of the experiment averaged 15 min, with an average duration of 2 min 15 s per block. The activity was administered individually in a quiet room with no visual or noise distractions.

The dependent variables were reaction time (RT), described as the amount of time it took (in ms) for children to press the response button after the visual stimulus appeared, and the accuracy of the responses. Both measures were obtained using E-Prime 3.0 software. Median response times and percent accuracy were calculated for each of the five blocks for each child.

#### 2.2.2. Working Memory Task (Covariable)

Working memory was assessed using the non-verbal task “*Torpo el topo torpe/Torpo the clumsy mole*” from the Childhood Neuropsychological Assessment Test (original Spanish version: Test de Evaluación Neuropsicológica Infantil (TENI) [47]. This instrument, developed and validated in Chile, assesses working memory with a Cronbach’s reliability index of 0.8. The nonverbal task involves recalling a visual sequence; participants observe a mole appearing in different holes in a 3 × 3 grid on a tablet screen. They are told that the mole is lost and is testing the holes to find an exit. The mole appears in a sequence of two holes, and after a bell sounds, the child must repeat the order of appearance. The sequence increases to eight positions. Practice trials are provided and repeated until the child fully understands the instructions before proceeding to the test. The task ends when the child fails two consecutive trials, and the analysis is based on the accuracy of the responses.

## 3. Results

All statistical analyses were carried out in SPSS 22. To ensure data reliability, outliers were removed using a criterion of two standard deviations above and below the mean. This approach effectively eliminated RT data points significantly deviating from the norm [48]. Approximately 7% of the RT data points in the entire sample were identified as outliers. Following data cleaning, a 2 (group: DLD, TD) × 5 (blocks: 1–5) mixed-design factorial ANOVA was employed to analyze reaction time and accuracy.

### 3.1. Reaction Time

#### 3.1.1. General Analysis RT

**Figure 2:**
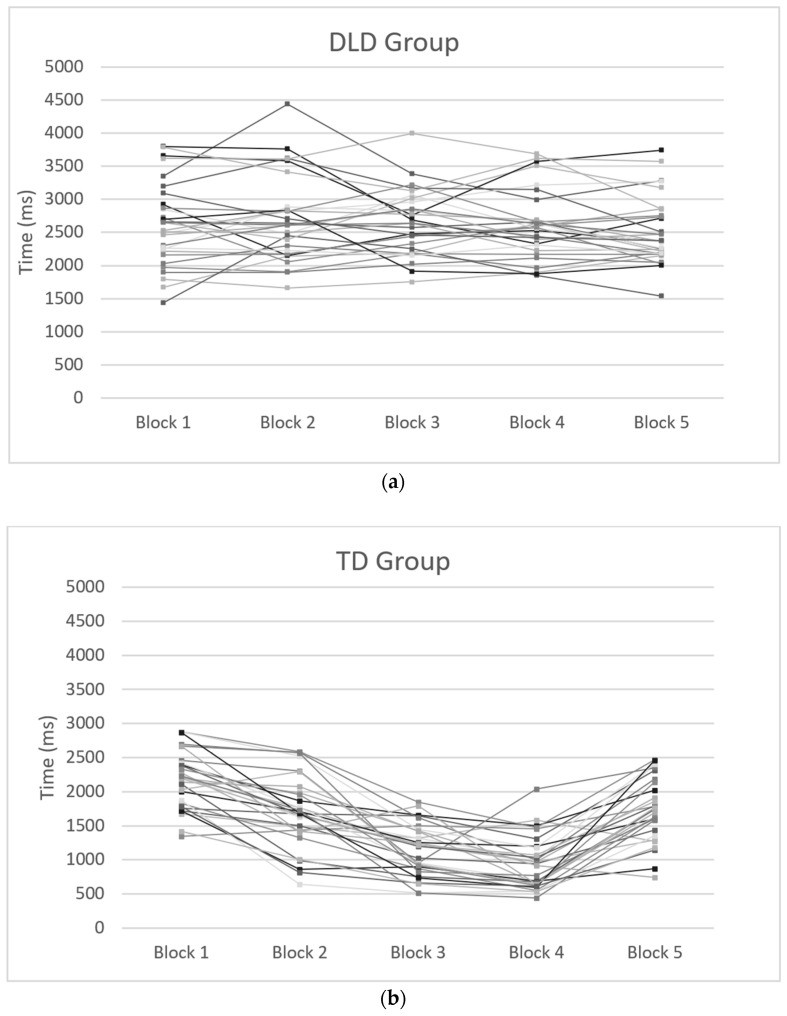
(**a**) DLD group mean RT (ms) by subject and block. (**b**) TD group mean RT (ms) by subject and block.

The results showed no significant main effects for either group or block. However, there was a notable interaction between group and block (F(4, 232) = 41.872, *p* < 0.001, η^2^partial = 0.419). This indicates that the DLD and TD groups exhibited different patterns of reaction time performance across the five blocks (Figure 2a,b). The observed power for this interaction was 1, suggesting a high likelihood of detecting significant effects. Independent sample *t*-tests indicated that the DLD group had significantly longer reaction times than the TD group in all blocks (block 1: t(58) = 2.974, *p* = 0.004; block 2: t(58) = 6.718, *p* < 0.001; block 3: t(58) = 12.838, *p* < 0.001; block 4: t(58) = 13.991, *p* < 0.001; block 5: t(58) = 5.712, *p* < 0.001).

After introducing the covariable of working memory into the model, the interaction effect between group and blocks remained statistically significant (F(4, 228) = 25,028, *p* = < 0.001, η^2^partial = 0.305). This suggests that the inclusion of performance in the task “Torpo the clumsy mole” did not result in a significant change in the interaction effect, indicating that the covariable did not have a substantial impact on the relationship for reaction time between the group and blocks.

When comparing both figures, greater individual differences were observed in the DLD group than in the TD group (see Figure 2a). Individual statistical analyses were conducted to observe the behavior of subgroups in the sample using t-tests between block 1, when the sequencing training begins, and block 4, when the training concludes. It was found that 56.6% of the DLD group did not experience changes throughout the task, 23% increased their reaction times, and only 20% decreased their reaction time, demonstrating more efficiency in the task. In contrast, in the control group, with the same analysis procedure, 93.3% decreased their reaction time, showing greater efficiency in the task; 6.6% did not experience significant changes; and no subject increased their reaction times (see Figure 2b).

#### 3.1.2. Group Analysis RT

In the DLD group, no significant effects were observed between blocks (F(4, 116) = 1.152, *p* = 0.336, η^2^partial = 0.038). These results suggest that no implicit learning took place in the DLD group, as indicated by the SRT task paradigms of procedural memory [27]. When introducing the covariable of working memory into the model, there remained no significant effects remained (F(4, 112) = 1.327, *p* = 0.264, η^2^partial = 0.045).

In the TD group, statistically significant effects between blocks were observed (F(4, 116) = 77.128, *p* < 0.001, η^2^partial = 0.727), indicating a progressive decrease in reaction times across blocks 1–4. Furthermore, there was a significant difference between the final serial pattern block (block 4) and the random block (block 5), where the TD group’s reaction time increased (t(29) = −9.548, *p* < 0.001). When introducing the covariable of working memory into the model, the effect in the blocks remained significant (F(4, 112) = 4.341, *p* < 0.005, η^2^partial = 0.045).

### 3.2. Accuracy

#### 3.2.1. General Analysis Accuracy

**Figure 3:**
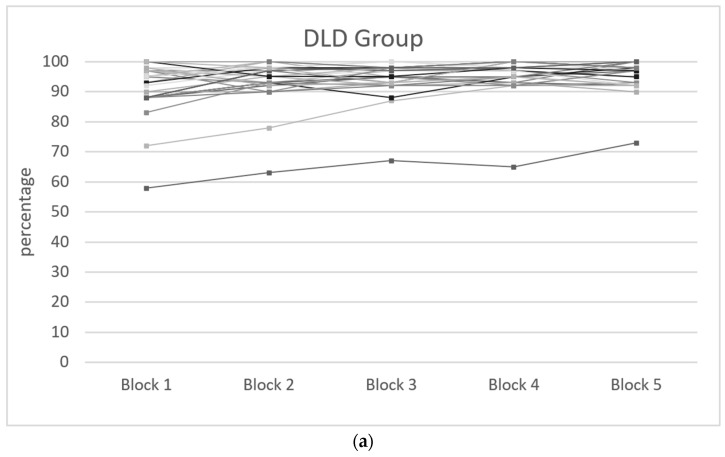

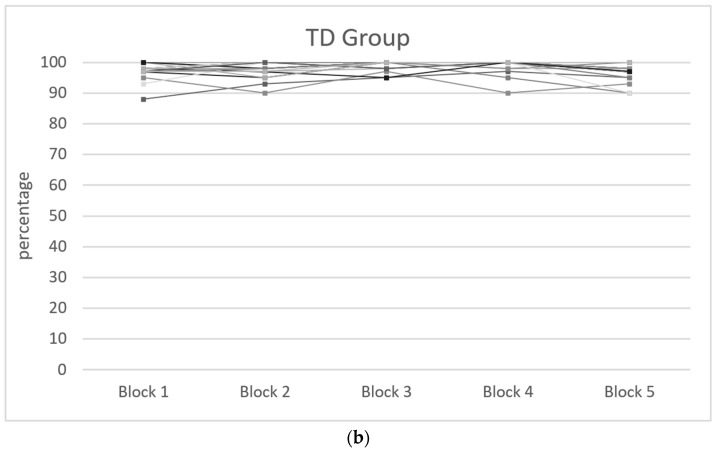
(**a**) DLD group mean accuracy (percentage) by subject and block. (**b**) TD group mean accuracy (percentage) by subject and block.

The analysis of accuracy revealed no significant main effect for either group or block but a significant interaction between group and block (F(4, 232) = 7.780, *p* < 0.001, η^2^partial = 0.118), indicating different patterns of accuracy performance in the DLD and TD groups across the five blocks. The observed power for this interaction was 0.99, suggesting a high likelihood of detecting significant effects. Independent sample t-tests indicated that the DLD group exhibited a significantly lower level of accuracy compared to the TD group in the serial pattern blocks (block 1: t(58) = −4.261, *p* < 0.001; block 2: t(58) = −3.359, *p* < 0.001; block 3: t(58) = −4.263, *p* < 0.001; block 4: t(58) = −3.044, *p* < 0.001). However, the difference between the groups in the random block was not statistically significant (block 5: t(58) = −1.740, *p* = 0.087) (Figure 3a,b).

After introducing the covariable of working memory into the model, the interaction effect between group and blocks remained statistically significant (F(4, 228) = 5795, *p* = < 0.001, η^2^partial = 0.092). This suggests that the inclusion of performance in the task “Torpo the clumsy mole” did not result in a significant change in the interaction effect, indicating that the covariable did not have a substantial impact on the relationship for accuracy between the group and blocks.

Bellow, the frequency tables of the participants are displayed.

Table 2 and Table 3 show the mean values as well as the lower and upper ranges for both groups. As can be observed, the minimum values in the DLD children were much lower than the minimum values of the TD group. However, in both the DLD and the TD children, the skewness index was negative, indicating that both populations exhibited similar behavior, with a predominantly rightward distribution. In this distribution, there were a few participants with low performance, some with acceptable performance, quite a few children with notable performance, and some children with outstanding performances. These results could suggest a ceiling effect associated with the task, especially in DLD children. Thus, the overall results can be understood in terms of efficiency, wherein the TD group took less time to succeed compared to the DLD group, whose successes involved greater effort [49].

#### 3.2.2. Group Analysis Accuracy

In the DLD group, there was a significant main effect for block (F(4, 116) = 10.036, *p* < 0.001, η^2^partial = 0.257), indicating a progressive increase in accuracy performance across all blocks. However, this difference was not significant between blocks 4 and 5 (t(29) = −0.604, *p* = 0.551). This suggests that the children became more efficient with the task but were not affected by the serial pattern. When introducing the covariable of working memory into the model, the effects were no longer significant (F(4, 112) = 1.161, *p* = 0.332, η^2^partial = 0.040).

In the TD group, there were statistically significant results (F(4, 116) = 4.774, *p* < 0.001, η^2^partial = 0.141), indicating differences in accuracy across blocks. Furthermore, there was a significant difference between the final serial pattern block (block 4) and the random block (block 5), where the level of accuracy decreased (t(29) = −9.548, *p* < 0.001). These results suggest that the TD group demonstrated learning in the serial pattern blocks, with an increase in accuracy up to the fourth block, followed by an impact on the fifth block due to its random nature, leading to a decrease in the accuracy percentage. When introducing the covariable of working memory into the model, the effects were no longer significant (F(4, 112) = 0.782, *p* = 0.539, η^2^partial = 0.027).

The covariable of working memory appeared to modify the significance of the observed effects, suggesting that working memory might play a role in explaining variations in accuracy performance in both the DLD and the TD group.

### 3.3. Correlations

To investigate potential correlations between language performance in the TELD-3: S and procedural memory, Pearson’s correlation coefficient (Pearson R) was employed. No correlations were found in reaction time for either the DLD or the TD group. However, in terms of the percentage of accuracy, correlations were observed exclusively within the DLD group, spanning from block 1 to block 4 (see Table 4). Notably, no correlation was identified in the random block. These findings suggest a significant linear relationship between language performance in the TELD-3: S and the accuracy of responses in the procedural memory task, specifically during the sequential pattern blocks (blocks 1–4).

## 4. Discussion

The current study aimed to explore sequence learning as a function of procedural memory in Spanish-speaking preschool children diagnosed with DLD. The PDH [13,14,15] raises two central ideas: One is the separation between the two memory systems of procedural and declarative memory, where the mechanism that fails is the procedural one, while the declarative system remains intact [13]. The other relevant assumption is that there is a close association between implicit learning and grammatical performance. We discuss our results based on the two assumptions from the PDH proposal.

Firstly, regarding the assumption of procedural memory, our evidence points to a deficit in sequence learning in the DLD population rather than exhibiting a general deficiency in procedural learning ability [22]. In terms of RTs, the significant interaction between group and block revealed distinct performance patterns across the five blocks for the two groups. The significantly longer RTs consistently observed in the DLD group compared to the TD group across all blocks underscore the substantial differences in their processing speed and learning trajectories. Additionally, the DLD group’s lack of significant RT differences between blocks suggests that children with DLD did not implicitly grasp the sequence, which is indicative of a deficit in this function of procedural memory. 

In contrast, the TD group demonstrated a progressive decrease in reaction times across blocks 1 to 4, which is indicative of an enhanced efficiency in processing the serial pattern. Of particular significance, a substantial difference emerged between the final serial pattern block (block 4) and the random block (block 5), resulting in an increase in reaction time. 

As for the individual differences reported in reaction times, they were present in the DLD group, where only 20% of the children showed an improvement in performance. This population is described as heterogeneous, and this was demonstrated in the different performance patterns in the SRT task compared to the TD group. 

Our results point to the difficulty faced by children with DLD in processing and in responding to stimuli presented in the SRT task. Likewise, our study showed the good performance of the TD group, which is in line with the notion that the functions of procedural memory seem to be preserved in early stages in Spanish-speaking preschool children. These results corroborate prior studies conducted on English-speaking populations [22,26,27], in which the same difficulties were found in DLD children compared to their TD peers in children between 5 and 10 years. In the case of Romance languages, a similar result was found when the authors introduced a more complex sequence of 12 items in the SRT task in DLD children aged between 7 and 13 years old compared to their TD counterparts [30].

On the contrary, the evidence reported in some of the experiments by Gabriel et al. [29,30,32] found no significant differences between DLD and TD children in sequential learning. However, those results can be explained by two factors: Firstly, the number of sequences was reduced, and secondly, the age of the subjects was higher (6 to 13 years). The experimental manipulation differences in this study involved a lower task difficulty for children with better performance due to the maturation of this procedural function. 

In the same vein, West et al. [50], in a meta-analysis study, criticize the assumptions of procedural skills in the DLD population based on the argument that performance in the SRT task can become conscious and not necessarily have an association with procedural memory. The authors also initiate a discussion about the presumed causality of procedural memory in the symptomatology of DLD or whether this deficit is rather a consequence of this disorder. Finally, the authors argue whether the motor skills of the subjects are deficient and whether this would affect their overall performance in the procedural memory task. It is necessary to incorporate new cognitive tasks to investigate the conscious or causal elements of procedural memory of children with DLD. However, when including the working memory task, the results in reaction times persisted in both groups, suggesting that sequential learning goes beyond motor learning at least in reaction times.

These results contrast with those of Jackson et al. [38], who compared procedural, declarative, and working memory performance between children with DLD and their TD peers. They found that the deficit in working memory could largely account for the impaired declarative and procedural memory. In their case, the investigators used three different working memory tasks, with only one of them being visuospatial. Therefore, the results may be related to the verbal aspect of working memory, which was not assessed in our investigation.

Regarding accuracy, a high level of accuracy was observed in both groups, suggesting that the individuals understood the task. The subsequent analysis revealed a significant interaction between group and block. In blocks 1 to 4, the DLD group exhibited a significantly lower level of accuracy compared to the TD group, indicating a major challenge in accurately responding to the stimuli associated with the procedural memory task. No significant difference was found in the random block, suggesting that the DLD group’s performance was similar to that of the TD group when the sequential pattern was not involved. This lack of difference in the random block could be attributed to a dual effect: The DLD group exhibited an ascending performance trend over time, potentially compensating for the impact of the random sequence, while concurrently, the TD group displayed a decrease in accuracy in the final block, possibly due to the unexpected shift from a learned pattern to a random one.

In the DLD group, a significant main effect for block was observed, indicating a progressive increase in accuracy performance across all blocks. However, there was no significant difference in accuracy between blocks 4 and 5, suggesting that the children became more efficient with the task but were not influenced by the serial pattern. This outcome is not well documented in previous studies [11,26,27,28], which mainly focus on the analysis of reaction times. 

In contrast, the TD group exhibited significant accuracy disparities across blocks, notably, a reduction between the final serial pattern block (block 4) and the random block (block 5). This drop in accuracy could be attributed to participants’ difficulty in adapting to the random presentation of stimuli, an occurrence anticipated within the SRT task framework of procedural memory [15,22,51].

A potential explanation for the divergence in the DLD group could be the nature of the SRT task, where participants are required to rapidly respond to stimuli by pressing a button, involving motor skills encompassing nonoral movements (e.g., hand movements). Considering that both oral and nonoral motor sequencing skills are recognized to be impaired in DLD [14,52], it is conceivable that these skills show improvement through repetitive training [11]. 

The potential motor learning observed in the DLD group in successful trials may be attributed to the phases of motor simulation and execution, which could have favored the DLD group according to the assumptions of embodied theories [53,54,55,56]. In an exploratory study under a paradigm of induced plasticity in children comparing DLD and TD children [57], the authors found that transfer movement in the induced plasticity technique resulted in facilitation rather than interference in children with DLD compared to TD children. In the DLD group, induced plasticity caused a kind of motor training capable of activating the involved motor areas without saturating them as expected. However, the inclusion of working memory as a covariable modulated the main effects of both groups. These results should be taken with caution, as when exploring individual differences in asymmetry tasks, a ceiling effect was found in the accuracy percentages in both groups. Consequently, the modulation of working memory only serves to confirm this ceiling effect, since both tasks involve a visuospatial response.

We will now discuss the second assumption regarding the relationship between linguistic skills and implicit learning. The examination of potential correlations between language performance, as measured by the TELD-3: S, and procedural memory in children with DLD yielded interesting findings. Although no correlations were found in terms of reaction time, a notable and statistically significant linear relationship emerged in the DLD group concerning the percentage of accuracy during the sequential pattern blocks (blocks 1-4) of the SRT task. These correlations suggest that as language proficiency, as assessed by the TELD-3: S, improves in children with DLD, there is a corresponding enhancement in their accuracy in responding to the sequential patterns presented in the procedural memory task. This finding underscores the interconnectedness of linguistic abilities and procedural memory, particularly in the context of DLD. The absence of correlation in the random block (block 5) further underscores the specificity of this relationship to situations requiring sequential pattern recognition. Correspondingly, Hsu and Bishop [22] assessed procedural learning in three groups: one group with DLD children, a second with TD peers, and a third group of younger children matched by grammar level with the DLD group. Performance was assessed using three tasks: the SRT task, a task evaluating verbal sequential learning (Hebb effect), and a motor procedural task that did not involve sequencing (pursuit rotor). The results indicated lower performance in DLD only in the SRT task, where children with DLD exhibited performance similar to that of the grammar-matched group and poorer results than age-matched controls. This suggests that language development, particularly grammatical abilities, may be linked to sequential pattern recognition implicated in procedural memory.

The linguistic abilities assessed by the Receptive TELD-3: S include lexical knowledge, understanding of specific words, knowledge of conceptual categories, and aspects of grammar encompassing both syntax and morphology but in general terms. Further investigation of these correlations with specific tasks assessing pure grammatical aspects is necessary to understand their relationship with procedural learning.

## 5. Conclusions

In conclusion, the present study introduces novel evidence on the role of procedural memory in children with DLD as well as TD children using an experimental paradigm at an early age, a context unexplored in previous studies focusing on the Spanish language acquisition process. These results serve as evidence of sequential learning implicating procedural memory in individuals with DLD as well as the relationship between linguistic aspects and the sequential learning of procedural memory. Our results align with previous evidence that considers age as a relevant factor in the development of some functions of procedural memory, as well as the repetitive practice of complex 10-item sequences [11]. 

The inclusion of working memory as a covariable aimed to explore whether working memory deficits could account for observed procedural memory differences. However, the results indicate that working memory did not substantially impact the relationship between group and procedural memory since it did not affect the reaction time interaction with block and group, although it did modulate accuracy. The type of memory used was visuospatial, a type of memory little explored in previous research [38]; however, it would be interesting to include a verbal type of memory to reach more specific conclusions about the modality of the task.

Considering these research insights, the understanding of the role of procedural memory in DLD carries practical implications for the development of language intervention and early stimulation strategies. By focusing on enhancing procedural memory skills, these interventions have the potential to mitigate linguistic limitations and enhance language outcomes for individuals with DLD.

Some limitations of the study include the lack of assessment of other cognitive abilities, such as declarative memory and motor skills, as well as sample control based on IQ. The use of other modalities in the task, such as verbal, highlights avenues for future research. A broader range of tasks, age groups, and memory systems should be investigated to provide a more comprehensive understanding. Furthermore, linguistic tasks of a morphosyntactic nature alongside a procedural memory task could explore more directly the relationship between cognitive and linguistic aspects involved in children with DLD. As language intervention and early stimulation strategies are developed, these insights can be instrumental in alleviating linguistic limitations and enhancing language outcomes for individuals with DLD. 

## Figures and Tables

**Table 1:** Participant characteristics.

Variable	DLD (*n* = 30)			TD (*n* = 30)			Comparison
	M	SD	Min–Max	M	SD	Min–Max	
Age (months)	55.03	3.85	48–60	56.17	2.52	49–60	t = −1.35 *p* = 0.18
TELD3-S rec	82.06	5.36	68–91	100.8	5.59	91–112	t = −13.24 *p* ≤ 0.001
TELD3-S exp	82.47	5.85	67–93	96.3	5.97	82–109	t = −9.06 *p* ≤ 0.001
TELD3-S total	164.53	3.48	156–169	197.1	5.42	185–204	t = −27.682 *p* ≤ 0.001

Note: Group mean values (M), standard deviation (SD), and score from TELD3-S.

**Table 2:** Measures of central tendency in accuracy for the DLD group.

	DLD Group				
	Block 1	Block 2	Block 3	Block 4	Block 5
Mean	91.233	93.600	94.433	95.367	95.733
Standard error of the mean	1.588	1.318	1.111	1.161	0.971
Standard deviation	8.696	7.218	6.084	6.359	5.317
Skewness	−2.287	−3.062	−3.375	−3.943	−2.836
Standard error of skewness	0.427	0.427	0.427	0.427	0.427
Minimum	58	63	67	65	73
Maximum	100	100	100	100	100

**Table 3:** Measures of central in accuracy tendency for the TD group.

	TD Group				
	Block 1	Block 2	Block 3	Block 4	Block 5
Mean	98.133	98.267	99.300	99.133	97.633
Standard error of the mean	0.317	0.439	0.263	0.428	0.499
Standard deviation	1.737	2.406	1.442	2.345	2.735
Skewness	−0.981	−1.881	−2.120	−3.008	−1.590
Standard error of skewness	0.427	0.427	0.427	0.427	0.427
Minimum	93	90	95	90	90
Maximum	100	100	100	100	100

**Table 4:** Correlation between block accuracy and TELD3-s.

Variable	DLD Accuracy					TD Accuracy				
	Block 1	Block 2	Block 3	Block 4	Block 5	Block 1	Block 2	Block 3	Block 4	Block 5
TELD-3: S rec	0.431 *	0.399 *	0.399	0.407 *	0.348	0.070	−0.121	−0.236	0.005	0.128
TELD-3: S exp	−0.233	−0.213	−0.089	−0.164	−0.162	0.059	0.273	0.001	0.196	0.026
TELD-3: S total	0.271	0.257	0.371 *	0.351	0.263	0.138	0.175	−0.242	0.221	0.161

Note: * = Indicates significant differences.

## Data Availability

The data generated and analyzed in this study are available on reasonable request from the corresponding author. The data are not publicly available as they are human data from adults and children in neurotypical and clinical groups.
